# Predictive Radiomic Models for the Chemotherapy Response in Non-Small-Cell Lung Cancer based on Computerized-Tomography Images

**DOI:** 10.3389/fonc.2021.646190

**Published:** 2021-07-07

**Authors:** Runsheng Chang, Shouliang Qi, Yong Yue, Xiaoye Zhang, Jiangdian Song, Wei Qian

**Affiliations:** ^1^ College of Medicine and Biological Information Engineering, Northeastern University, Shenyang, China; ^2^ Key Laboratory of Intelligent Computing in Medical Image, Ministry of Education, Northeastern University, Shenyang, China; ^3^ Department of Radiology, Shengjing Hospital of China Medical University, Shenyang, China; ^4^ Department of Oncology, Shengjing Hospital of China Medical University, Shenyang, China; ^5^ Department of Electrical and Computer Engineering, University of Texas at El Paso, El Paso, TX, United States

**Keywords:** lung cancer, radiomics, CT images, chemotherapy response, machine learning

## Abstract

The heterogeneity and complexity of non-small cell lung cancer (NSCLC) tumors mean that NSCLC patients at the same stage can have different chemotherapy prognoses. Accurate predictive models could recognize NSCLC patients likely to respond to chemotherapy so that they can be given personalized and effective treatment. We propose to identify predictive imaging biomarkers from pre-treatment CT images and construct a radiomic model that can predict the chemotherapy response in NSCLC. This single-center cohort study included 280 NSCLC patients who received first-line chemotherapy treatment. Non-contrast CT images were taken before and after the chemotherapy, and clinical information were collected. Based on the Response Evaluation Criteria in Solid Tumors and clinical criteria, the responses were classified into two categories: response (n = 145) and progression (n = 135), then all data were divided into two cohorts: training cohort (224 patients) and independent test cohort (56 patients). In total, 1629 features characterizing the tumor phenotype were extracted from a cube containing the tumor lesion cropped from the pre-chemotherapy CT images. After dimensionality reduction, predictive models of the chemotherapy response of NSCLC with different feature selection methods and different machine-learning classifiers (support vector machine, random forest, and logistic regression) were constructed. For the independent test cohort, the predictive model based on a random-forest classifier with 20 radiomic features achieved the best performance, with an accuracy of 85.7% and an area under the receiver operating characteristic curve of 0.941 (95% confidence interval, 0.898–0.982). Of the 20 selected features, four were first-order statistics of image intensity and the others were texture features. For nine features, there were significant differences between the response and progression groups (*p* < 0.001). In the response group, three features, indicating heterogeneity, were overrepresented and one feature indicating homogeneity was underrepresented. The proposed radiomic model with pre-chemotherapy CT features can predict the chemotherapy response of patients with non-small cell lung cancer. This radiomic model can help to stratify patients with NSCLC, thereby offering the prospect of better treatment.

## Introduction

According to the Global Cancer Incidence and Mortality Report in 2018, lung cancer was the most commonly diagnosed cancer (11.6% of all cases) and the leading cause of cancer deaths (18.4% of all cancer deaths) (1, 2), with non-small cell lung cancer (NSCLC) accounting for 80% to 85% of all lung cancers. However, despite considerable advances in diagnosis and treatment over the years, the 5-year survival rate of lung-cancer patients is currently less than 18% (54% for localized-stage disease, 26% for regional stage, and 4% for distant stage) (3–5). As reported, 74% of cases are diagnosed at the regional or distant stage (3), and any patient diagnosed as being at stage IIIA or IV is virtually unresectable and has no choice but to receive radiotherapy or chemotherapy with severe side effects.

The heterogeneity and complexity of NSCLC tumors mean that NSCLC patients at the same stage can have different chemotherapy prognoses (6). According to the Response Evaluation Criteria in Solid Tumors (RECIST), treatment responses can be divided into four types: (i) complete response (CR), (ii) partial response (PR), (iii) progressive disease (PD), and (iv) stable disease (SD) (7). However, there are currently few quantitative criteria or models that can predict the NSCLC chemotherapy response from pre-treatment information (8). Accurate predictive models could recognize NSCLC patients likely to respond to chemotherapy so that they can be given personalized and effective treatment.

Radiomics is a potential bridge between medical imaging and personalized medicine (9, 10). In this approach, artificial intelligence is used to convert image data from a lesion region into a high-dimensional feature space and to construct predictive models for various clinical outcomes (11, 12). Radiomics has been used successfully in biological oncology for detection, differential diagnosis, phenotype or subtype stratification, prognosis prediction, and even the prediction of invasiveness and gene mutation status (13–17).

Radiomics has achieved exceptional results in predicting the prognosis of NSCLC treatment with survival as the endpoint. For example, based on a dataset of 1194 NSCLC patients treated with either radiotherapy or surgery, Hosny et al. constructed convolutional neural network models that could predict 2-year overall survival from pre-treatment computerized-tomography (CT) images with an accuracy of 70% (18). For 179 stage-III NSCLC patients treated with definitive radiotherapy and chemotherapy, Xu et al. designed a deep-learning model using time-series CT scans and found that it was significantly predictive of survival and cancer-specific outcomes (4). Wang et al. collected CT images and clinical information for 173 NSCLC patients and trained a radiomic model that could predict the range of a patient’s prognosis survival time (6). Song et al. established a Cox regression model with a least absolute shrinkage and selection operator for CT images to predict the progression-free survival of stage-IV epidermal growth factor receptor (EGFR)-mutated NSCLC patients being treated with EGFR tyrosine kinase inhibitors (19). Paul et al. used a transfer-learning model to extract deep features to predict short-and long-term survivors with lung adenocarcinoma with an accuracy of 90% (20). Lou et al. used deep-learning methods of pre-treatment CT scans to analyze survival and found an individualized radiation dose that gave an estimated probability of treatment failure of below 5% (21).

Moreover, predicting the chemotherapy response in NSCLC earlier in the course treatment is very useful and promising. It can help clinicians make decisions on whether to adapt, intensify, or alter treatment plans early and improve patient outcomes (22). Compared with the long-term endpoint of survival, treatment response is a short-term prognosis endpoint that may help to identify precisely those NSCLC patients who are likely to benefit from chemotherapy.

However, to the best of our knowledge, few predictive models use chemotherapy response in NSCLC as the endpoint. Chen et al. proposed a radiomic model to predict NSCLC lesions shrinkage during treatment with either pembrolizumab or combinations of chemotherapy and pembrolizumab (23). The model used features extracted from lesions, margins, and blood vessels and reached an area under the curve (AUC) of 0.73 in a test cohort with 176 patients. Seki et al. had demonstrated the usefulness of CT and Positron Emission Tomography (PET)/CT in the early prediction of chemoradiotherapy in NSCLC (24). In the present study, we constructed a radiomic model that used pre-chemotherapy CT images to predict the NSCLC chemotherapy response.

## Materials and Methods

### Data Acquisition

**Figure 1:**
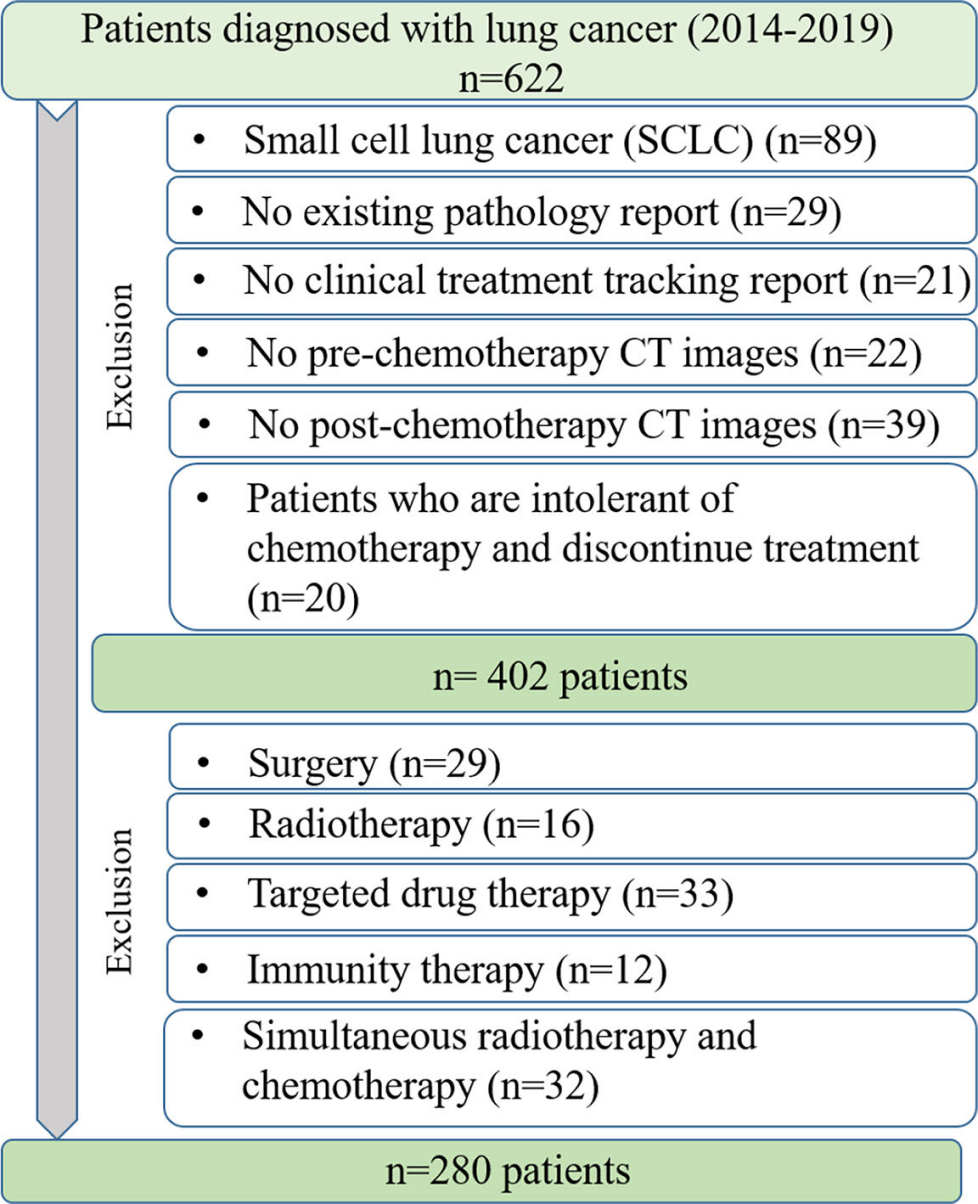
Criteria for data acquisition.

We enrolled 622 patients with lung cancer being treated at Shengjing Hospital of China Medical University between 2014 and 2019. The parameters for CT images acquisition are listed in **Table 1**. As shown in **Figure 1**, after two steps of exclusion criteria, 280 patients were included in our study. Their clinical characteristics are given in **Table 2**. This study was approved by the ethics committee of Shengjing Hospital of China Medical University and the waived informed consent forms were waived because it is a retrospective study.

**Table 1:** Parameters for CT image acquisition.

Parameter	Value
kVp (kV)	120
X-ray tube current (mean ±S.D.) (mA)	215.274 ± 70.816
Slice thickness (mm)	2.5 (*n =* 14); 3.0 (*n =* 244); 5.0 (*n =* 22)
Pixel size (mm)	0.783 ± 0.074
CT scanner manufacturer	GE Medical (*n =* 10), Siemens (*n =* 11), Toshiba (*n =* 13), Philips (*n =* 246)

**Table 2:** Clinical characteristics of NSCLC patients.

Characteristics			Response group	Progression group	*p* value
Number of patients			145	135	–
Gender		Male		79	77	0.605
		Female		66	58	
Age(years)			63.864 ± 10.042	64.402 ± 9.713	0.437
Histological type	Adenocarcinoma		119	109	0.201
	Squamous cell carcinoma		26	26	
Smoking status	Ever		49	74	0.002
	Never		96	61	
Number of treatment courses			4.492 ± 1.603	3.681 ± 1.396	<0.001
Chemotherapy drug	AP		53	36	–
	GP		29	34	
	TP		31	28	
	DP		32	37	

### Label of Treatment Response

NSCLC tumors were categorized according to RECIST jointly by an experienced radiologist and an experienced oncologist: (i) CR: disappearance of all target lesions, (ii) PR: at least 30% decrease in the sum of the diameters of the target lesions, (iii) PD: at least 20% increase in the sum of the diameters of the target lesions, and (iv) SD: neither sufficient shrinkage to qualify for PR nor sufficient increase to qualify for PD.

According to the requirement of clinical applications and radiologist’s advice, we had excluded the patients of SD in our study. The CR and PR patients were combined into a category named “response” and the PD patients were included into a category named “progression.” Finally, 145 NSCLC patients were labeled as response and 135 were labeled as progression.

### Overview of Study Procedure

**Figure 2:**
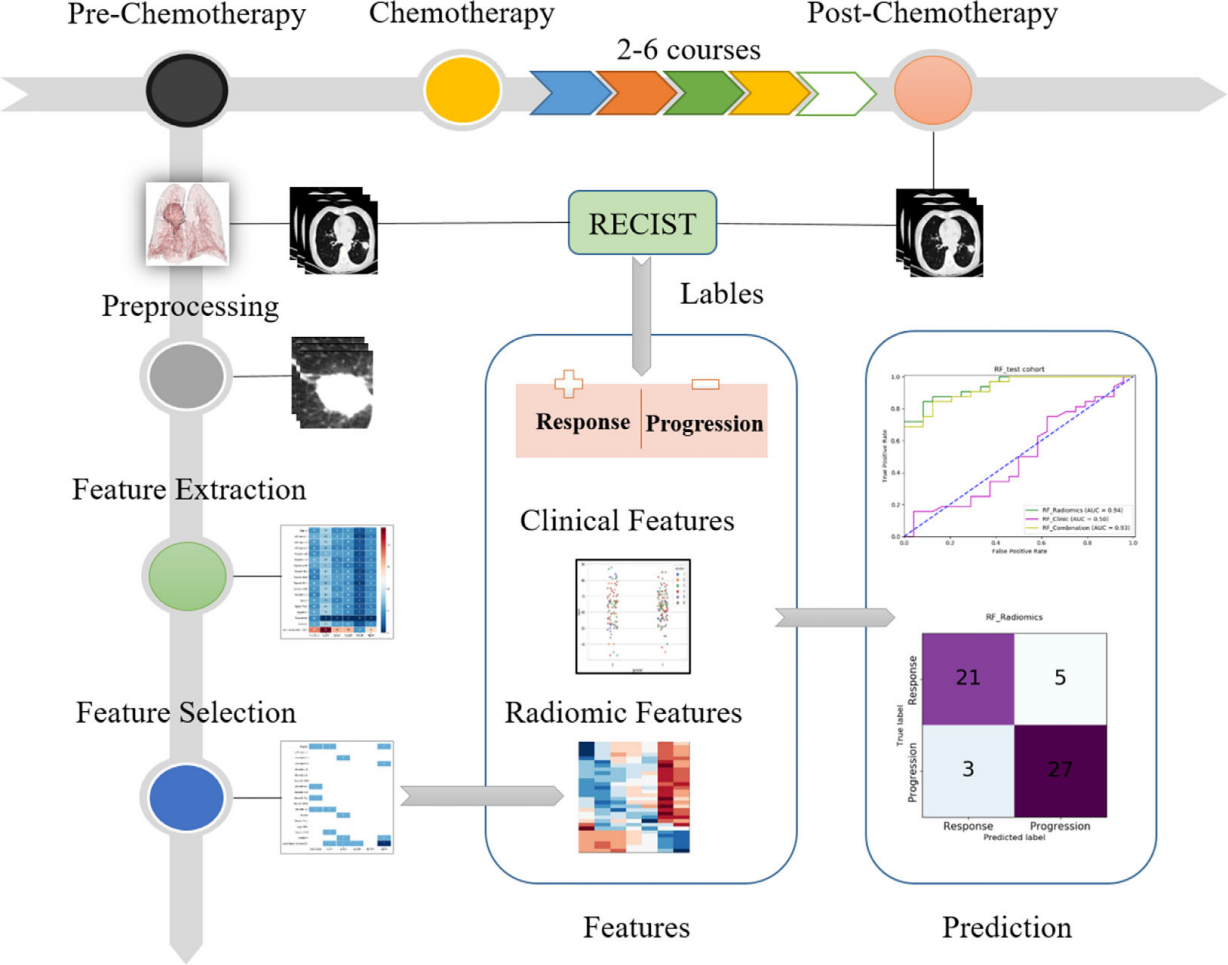
Flowchart of present study.

We split the total 280 patients into the training cohort (n = 224) and the independent test cohort (n=56). As shown in **Figure 2**, the study procedure had six steps. First, by comparing CT images taken before and after chemotherapy, responses were determined as being either response or progression. Second, in the preprocessing step, the tumor lesion in the pre-chemotherapy CT images was cropped to a cube. Third, radiomic features were extracted from the cropped cube. Fourth, discriminative features were selected with different methods and analyzed. Fifth, the selected radiomic features, labels, and clinical information were used to train the different models using the training cohort. Finally, the performance of the radiomic models was evaluated using the independent test cohort. The evaluation measures included the AUC of receiver operating characteristic (ROC) curve, confusion matrix, recall, precision, and F-score. The best cutoff value of ROC curve to calculate the confusion matrix and related measures was determined, whereas Youden index reach the maximum value.

### Feature Extraction

First, all the pre-treatment CT images for the patients were interpolated into voxels of 0.750 × 0.750 × 3.000 mm. To include the characteristic information [both the tumor lesion and its habitat (8, 12)], we cropped a 64 × 64 × 32 cube from the lesion center[determined by software 3D Slicer (25)]. In our study, this cube can include the largest lesion and no cube includes more than one lesion. Because all the patients are in the advanced stage and have taken chemotherapy, the lesion cannot be very small compared with the cube of 64× 64 × 32.

Then, we used the open-source Python package PyRadiomics to extract radiomic features from each cube (26). In total, 1,927 features were extracted from the original CT images, of which 1,629 meaningful features were used. Because the shapes and sizes were same for all cubes, the related features had no discriminative capability and were excluded (n=298).

It is should be noted that according to a previous study, besides the intra-tumor region, the extra-tumor marginal region may also provide predictive information for the treatment response and overall survival (8, 27, 28). Therefore, the features extracted from the cube in our study represent the characteristics not only of intra-tumor region but also of extra-tumor region.

### Feature Selection

Next, we used three algorithms to select discriminative features and passed them into the model for training and testing: random forest (RF) (29), mRMR (max-relevance and min-redundancy) (30), and relief (31). RF can be easily applied to select the critical features by ranking the importance score of features. It belongs to the embedded feature selection using SelectFromModel. Actually, the package of sci-kit learn has provided two ways of feature selection by using RF: (1) mean decrease impurity; (2) mean decrease accuracy. In our study, we directly used the way of “mean decrease accuracy.” Both mRMR and relief are the feature selection methods based on filter and publicly available (13).

Using the rule of thumb given by Gillies et al., with each feature corresponding to 10 samples in a binary classifier (12), we selected 20 features to represent each patient to do the next classification, and the performance of RF, mRMR, and relief for the feature selection was compared.

### Construction of Predictive Models

We selected three representative machine-learning classifiers: support vector machine (SVM), RF, and logistic regression (LR). We constructed a model to clarify the role of clinical information (gender, smoking status, age, pathology, course of treatment, and medicine), and we constructed another model with both clinical and radiomic features to assess whether that combination increased the predictive performance. Moreover, we also constructed a model with two clinical features of smoking status and course of treatment because there was a significant difference between the response and progression groups for these two features (**Table 2**). Correspondingly, a model with the combination of two significant clinical features and the selected radiomic features was constructed.

The optimal parameters of the model were determined by grid search technique and 10-fold cross-validation. Specifically, for each grid of parameters, the performance of the model was evaluated by the average of 10-fold cross-validation. The optimal parameters were determined after traversing all grids. During the 10-fold cross-validation, the training data were divided into 10 folds. For each of the 10 “folds,” a model was trained by using nine folds as the training data and validated by the remaining fold. With the determined optimal parameters, the model was retrained by all training data (n=224). Finally, the independent test cohort (n=56) was used to evaluate the retrained model and gave the performance measures. All these procedures were performed by strictly following the document given by the Sci-kit learn website (https://scikit-learn.org/stable/modules/cross_validation.html).

To find the optimal parameters in classification models, we used the grid search with cross-validation (GridSearchCV) to traverse the parameters within a certain range and with a specific interval. In SVM, the kernel parameter was set as “linear” or “rbf” (radial basis function); the parameter C ranged from 1 to 5 with the interval of 1; the gamma parameter was set as 0.125, 0.25, 0.5, 1, 2, or 4. Through the ten-fold cross-validation of the training cohort in each grid, the optimal value (or setting) of the kernel, C, and gamma were determined as “linear,” 3, and 1, respectively. In RF, n_estimators parameter ranged from 20 to 2000 with the interval of 10; max_features parameter was set as 2 or 3; min_sample_leaf ranged from 1 to 50 with the interval of 1. The optimal value of n_estimators, max_features, and min_sample_leaf was determined as 100, 3, and 2, respectively. In LR, C parameter ranged from 1 to 5 with the interval of 1; the penalty item was set as l1 or l2. By the same way, the optimal value of C and penalty item was determined as 3 and l1, respectively.

## Results

### Clinical Characteristics

**Figure 3:**
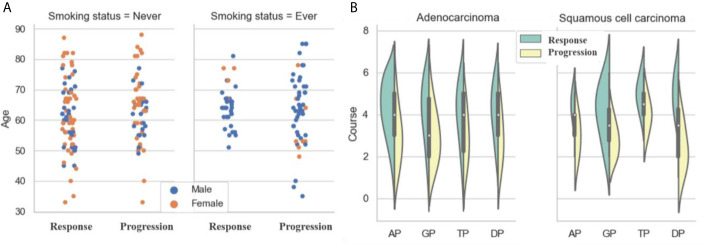
Analysis of clinical characteristics: **(A)** Statistics of ages, genders and smoking status; **(B)** Statistics of treatment courses and chemotherapy drugs.

As shown in **Figure 3A** and **Table 2**, there was no significant difference in gender between the response and progression groups. Similarly, there was no significant difference for age or histological type. For both groups, there were more patients with adenocarcinoma than with squamous cell carcinoma (119 *vs*. 26; 109 *vs*. 26). The progression group had a higher percentage of smokers than the response group [54.8% (74/135) *vs*. 33.8% (49/145)]. The response group had more treatment courses than the progression group (4.492 ± 1.603 *vs*. 3.681 ±1.396).

A platinum-based dual-drug regimen is the gold standard for the first-line treatment of advanced NSCLC. In our study, we included four common chemotherapy regimens: (i) AP: cisplatin or carboplatin combined with pemetrexed (n = 53 for response and n = 36 for progression), (ii) GP: cisplatin or carboplatin combined with gemcitabine (n = 29 for response and n = 34 for progression), (iii) TP: cisplatin or carboplatin combined with paclitaxel (n = 31 for response and n = 28 for progression), and (iv) DP: cisplatin or carboplatin combined with docetaxel (n = 32 for response and n = 37 for progression).

As shown in **Figure 3B**, for adenocarcinoma treated by AP, the response group had more patients than the progression group (47 *vs*. 27), but the opposite was the case for adenocarcinoma treated by GP (16 *vs*. 28). The situation for squamous cell carcinoma was the opposite of that for adenocarcinoma. For squamous cell carcinoma treated by AP, the response group had fewer patients than the progression group (6 *vs*. 9); for adenocarcinoma treated by GP, the response group had more patients than the progression group (13 *vs*. 6).

### Radiomic Characteristics

**Figure 4:**
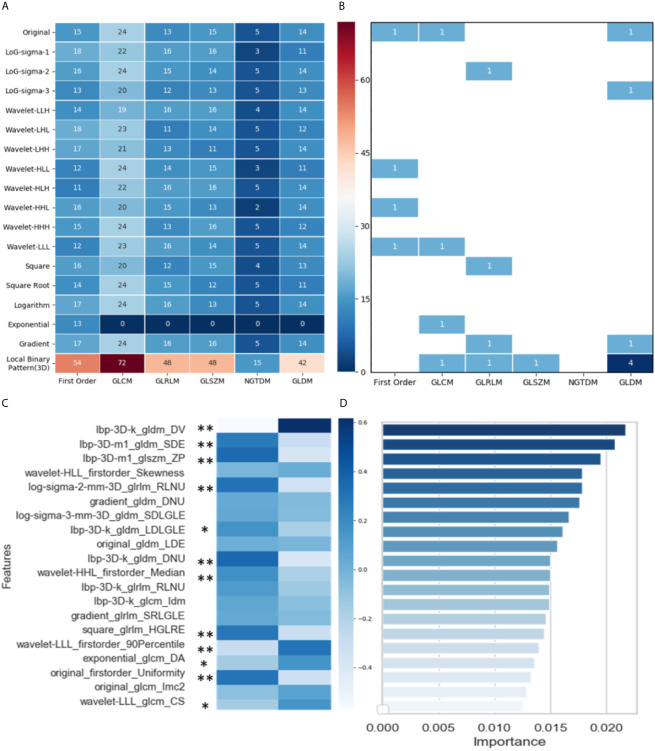
Analysis and selection of radiomic features: **(A)** Distribution of 1629 extracted features; **(B)** Distribution of 20 selected features; **(C)** Mean values of 20 highly informative features and significance analysis between two groups (* p < 0.05, ** p < 0.001); **(D)** Importance of 20 selected features.

**Figure 4A** shows the distribution of the 1,629 selected radiomic features. Of the six feature classes (columns), GLCM (gray-level co-occurrence matrix) had the most features (430/1629, 26.4%). Of the 18 filter classes (rows), local binary pattern (LBP) (3D) had the most features (279/1629, 17.1%).

Through three dimensionality reduction algorithms, the 20 most-informative features were selected from the 1629 radiomic features and input into the machine-learning classifiers. The distribution of these 20 features is shown in **Figure 4B**: gray-level dependence matrix (GLDM) had seven features, and first order, GLCM, and GLRLM (gray-level run-length matrix) each had four features. For the filter classes (rows), LBP (3D) had the most features (7/20, 35.0%). **Figure 4C** shows the mean values of these 20 highly informative features. In summary, 11 radiomic features differed significantly between the response and progression groups [nine features with *p* < 0.001 (**) and two features with *p* < 0.05(*)]. **Figure 4D** shows the importance of the 20 features selected *via* dimensionality reduction.

In the response group, the features small dependence emphasis (SDE), run length non-uniformity (RLNU), dependence non-uniformity (DNU), high gray level run emphasis (HGLRE), and uniformity are overrepresented, whereas dependence variance (DV) is underrepresented. SDE measures the distribution of small dependencies, with a larger value indicating less dependence and less-homogeneous textures. Similarly, larger values for RLNU and DNU indicate that there is less homogeneity among run lengths and dependencies in the image, respectively. DV measures the variance independence size in the image. Overall, the representation of these features indicates that NSCLC tumors in the response group are more likely to be heterogeneous in CT images than are those in the progression group.

### Dependence of Performance on the Feature Selection Method

**Figure 5:**
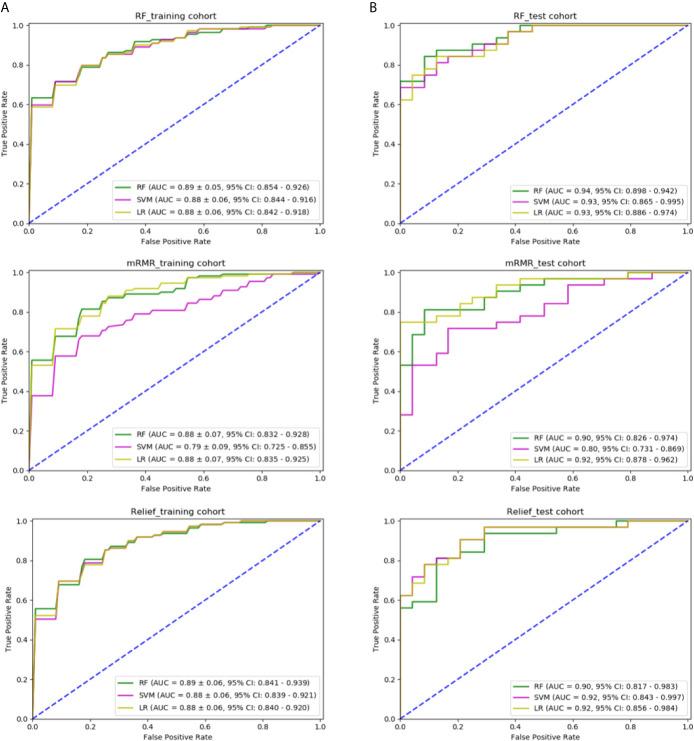
Comparison of predictive models with different classifiers and different methods of feature selection: **(A)** ROC curve of three models using features selected by RF, mRMR, and relief in the training cohort; **(B)** ROC curve of three models using features selected by RF, mRMR, and relief in the independent test cohort.

We tried three different feature selection methods, RF, relief, and mRMR, to clarify their impact on the classification results. In **Figure 5**, in the training cohort, for the feature selection method of RF, the AUC of RF, SVM, and LR classification models was 0.891 ± 0.05 (95% confidence interval (CI), 0.854–0.926), 0.882 ± 0.06 (95% CI, 0.844–0.916), and 0.883 ± 0.06 (95% CI, 0.842–0.918), respectively. For mRMR, the AUC was 0.886 ± 0.07 (95% CI, 0.832–0.928), 0.798 ± 0.09 (95% CI, 0.725–0.855), and 0.889 ± 0.07 (95% CI, 0.835–0.925), respectively. For relief method, the AUC was 0.890 ± 0.06 (95% CI, 0.841–0.939), 0.886 ± 0.06 (95% CI, 0.839–0.921), and 0.888 ± 0.06 (95% CI, 0.840–0.920), respectively. In the independent test cohort, for the feature selection method of RF, the AUC of RF, SVM, and LR classification models were 0.941 (95% CI, 0.898–0.942), 0.932 (95% CI, 0.865–0.995), and 0.935 (95% CI, 0.886–0.974), respectively. For mRMR, the AUC was 0.901 (95% CI, 0.826–0.974), 0.804 (95% CI, 0.731–0.869), and 0.923 (95% CI, 0.878–0.962), respectively. For relief method, the AUC was 0.902 (95% CI, 0.817–0.983), 0.921 (95% CI, 0.843–0.997), and 0.926 (95% CI, 0.856–0.984), respectively. The combination of the feature selection by RF and the classification model of RF generated the best predictive performance in both the training cohort and the independent test cohort.

### Performance of Machine-Learning Models

**Figure 6:**
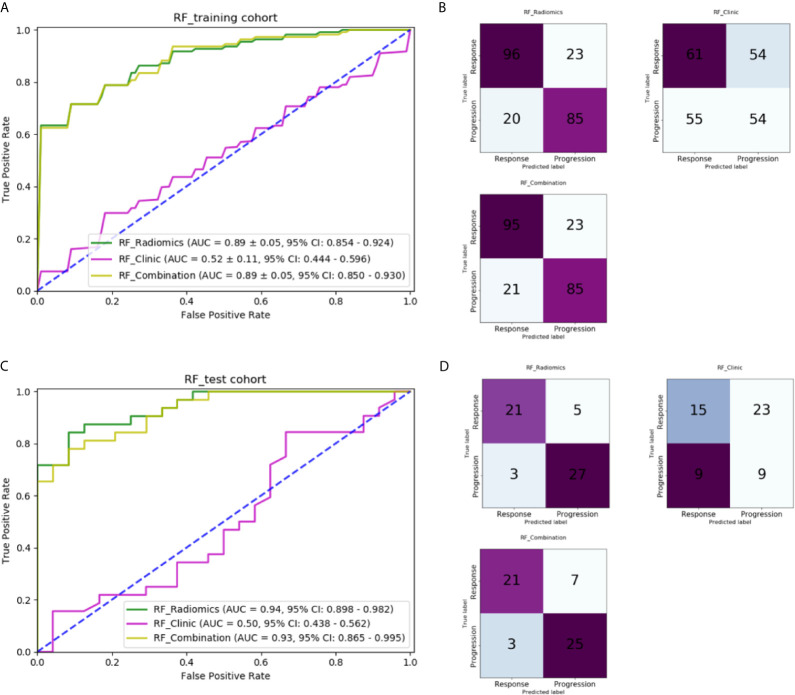
Comparison of machine-learning models: **(A)** ROC curves for different machine-learning models in the training cohort; **(B)** Confusion matrix of different machine-learning models in the training cohort; **(C)** ROC curves for different machine-learning models in the independent test cohort; **(D)** Confusion matrix of different machine-learning models in the independent test cohort.

**Table 3** lists the performance of the three machine-learning models, and **Figure 6** shows the receiver operating characteristic (ROC) curves and the areas under the curve (AUC). In the training cohort, the RF model with radiomic features had the best performance, its AUC was 0.891 ± 0.05 (95% CI, 0.854–0.924). In the independent test cohort, the RF model with radiomic features had the best performance. Its AUC was 0.941 (95% CI, 0.898–0.982), and its accuracy, recall, precision, and F-score were 85.7%, 0.875, 0.808, and 0.840, respectively. The cutoff of ROC curve was 0.438.

**Table 3:** Predictive performance of machine-learning models with radiomic features, clinical features, and combined features in the independent test cohort.

Classifier	Accuracy	AUC	Recall	Precision	F-score
RF_Radiomics	85.7%	0.941	0.875	0.808	0.840
RF_Clinic	42.9%	0.503	0.625	0.395	0.484
RF_Combination	82.1%	0.936	0.875	0.750	0.808

The RF model with five clinical features had an AUC of only 0.523 ± 0.11 (95% CI: 0.444–0.596) in the training and 0.503 (95% CI: 0.438–0.562) in the independent test cohort, which indicates that clinical features played hardly any role in predicting chemotherapy response in our study. The cutoff of ROC curve in the independent test cohort was 0.459.

The RF model with combined clinical and radiomic features did not perform better than the RF model with only radiomic features. The AUC of training cohort was 0.890 ± 0.05 (95% CI: 0.850–0.930). In the independent test cohort, the accuracy, recall, precision, and F-score of the former were 82.1%, 0.875, 0.750, and 0.808, respectively, which are lower than those of the RF model with only radiomic features. The AUC was 0.930 (95% CI: 0.865–0.995) with a cutoff of 0.543.

**Figure 7:**
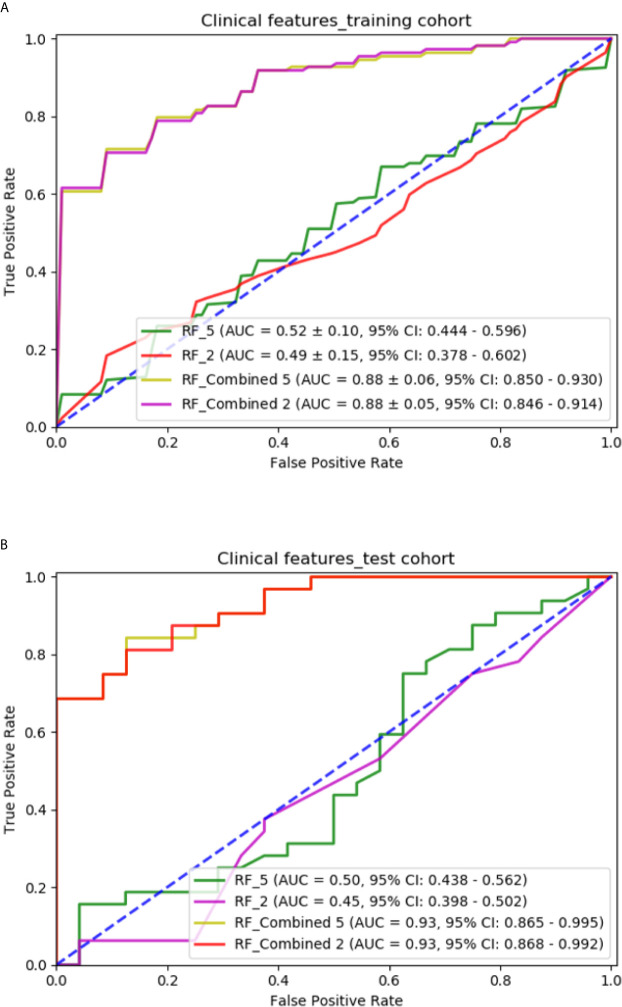
Comparison of predictive models with different clinical features: **(A)** ROC curves for different models in the training cohort; **(B)** ROC curves for different models in the independent test cohort.

The RF models with two significant clinical features are compared with those with five clinical features in **Figure 7**. The RF model with two significant clinical features had an AUC of 0.498 ± 0.15 (95% CI: 0.378–0.602) and 0.456 (95% CI: 0.398–0.502) in the training and independent test cohort, respectively. The RF model with the combination of two significant clinical features and the selected radiomic features had an AUC of 0.882 ± 0.06 (95% CI: 0.846–0.914) and 0.936 (95% CI: 0.868–0.992) in the training and independent test cohort, respectively. The performance of models with two significant clinical features was not as good as that of models with five clinical features.

### Performance for Different Chemotherapy Drugs and Histological Subtypes

**Table 4** presents the prediction accuracy of the RF model with 20 radiomic features for different chemotherapy drugs and histological subtypes. The prediction accuracy was higher for adenocarcinoma than squamous cell carcinoma (84.2% *vs*. 75.0%). A possible reason was the smaller number of patients with squamous cell carcinoma (*n* = 52). For chemotherapy drugs AP, GP, TP, and DP, the accuracy was 84.6% (77/91), 88.6% (62/70), 67.2% (41/61), and 87.9% (51/58), respectively. Of all eight combinations, the accuracy was highest at 93.8% (45/48) for adenocarcinoma treated by DP. The lowest accuracy was 54.5% for squamous cell carcinoma treated with TP; similarly, there were only 11 instances of this combination, which might have influenced the prediction.

**Table 4:** Prediction accuracy for different chemotherapy drugs and histological subtypes.

Chemotherapy drugs	Adenocarcinoma	Squamous cell carcinoma	Total
AP	85.2% (69/81)	80.0% (8/10)	84.6% (77/91)
GP	87.8% (43/49)	90.5% (19/21)	88.6% (62/70)
TP	70.0% (35/50)	54.5% (6/11)	67.2% (41/61)
DP	93.8% (45/48)	60.0% (6/10)	87.9% (51/58)
Total	84.2% (192/228)	75.0% (39/52)	–

## Discussions

### Clinical Characters

In this study, the progression group had a higher percentage of smokers than the response group, possibly indicating that NSCLC patients who smoke have a higher risk of progression during chemotherapy. Smoking is a high-risk factor for lung cancer (32, 33), and patients with lung cancer who continue to smoke after diagnosis can experience increased treatment-related toxicity and may have a decreased survival rate.

Another finding is that for the response group, a high percentage of those with adenocarcinoma were treated with AP and a high percentage of those with squamous cell carcinoma were treated with GP. This result agrees with the recommendation of AP for adenocarcinoma and GP for squamous cell carcinoma (34–37).

### Heterogeneity of Tumors

**Figure 8:**
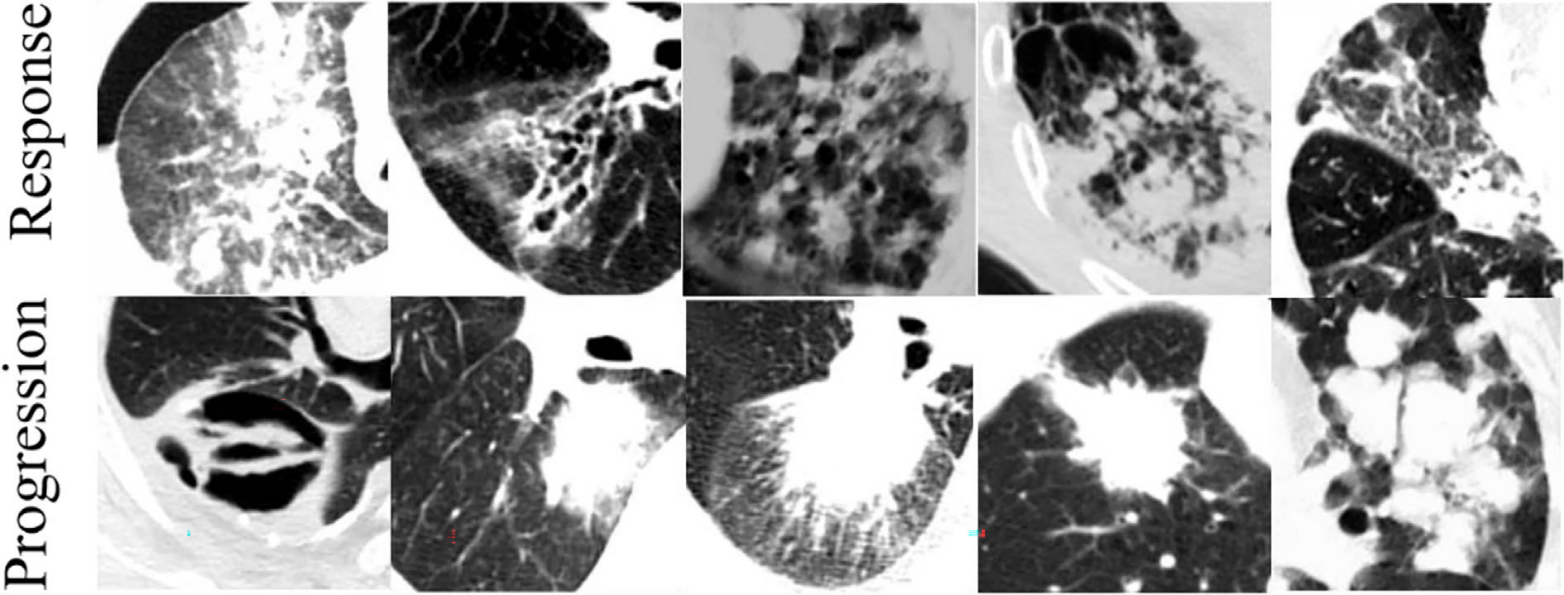
Typical CT images for response and progression groups.

One of our main findings is that NSCLC tumors in CT images are more heterogeneous in the response group than in the progression group. In the response group, the measures of heterogeneity (SDE, RLNU, and DNU) are overrepresented whereas the measure of homogeneity (DV) is underrepresented (**Figure 8**). This CT-driven textural heterogeneity may correlate with the tumor micro-environment heterogeneity, so the tumor growth rate, invasion ability, drug sensitivity, and prognosis will show differences in CT images (38). Imaging heterogeneity and micro-environment heterogeneity are important for therapeutic response, resistance, and clinical outcomes (39–41). NSCLC patients whose tumors have higher CT-driven textural heterogeneity have a longer overall mean survival (34.5 *vs*. 22.1 months) (42). Moreover, EGFR-mutated (EGFR+) lung adenocarcinoma is more heterogeneous than EGFR− in CT images (43).

For the cropped CT image cube used as the input in our study, the heterogeneity includes intra- and extra-tumor components. The information in the extra-tumor region has been considered to be useful for predicting the treatment response and overall survival (8, 27, 28). Extra-tumor heterogeneity emphasizes the contour between the tumor lesion and its habitat. Therefore, here, the higher heterogeneity in the NSCLC response group indicates the higher combination of intra-tumor texture heterogeneity and extra-tumor heterogeneity (the complexity of the tumor contour or shape).

### Advantage and Significance of Radiomic Model and Feature Selection

The RF model had an AUC of 0.941, and this test is simple, non-invasive, and quick. A predictive radiomic model could be used in the clinic before treatment to estimate the probability that a patient will respond to chemotherapy and high possibilities would give the oncologists more confidence in the chemotherapy, whereas otherwise other optional treatment plans should be considered.

We tried three different methods of feature selection to know which was suitable for our data and the RF method achieved the better performance than mRMR and relief. The possible reasons are given as follows: a) the mRMR algorithm does not provide a clear determination of the optimal amount of features and can thus still retain redundant features. b) relief is a filter-based feature selection method, but it is easy to ignore small samples and cannot reduce redundant features. We used RF feature selection method based on mean decrease accuracy strategy, it sorts the importance of features to find the most suitable feature subset (13, 44–46).

### Clinical Features Do Not Help Prediction

Of the five clinical features, smoking status and number of treatment courses differed significantly between the response and progression groups. Histological type and chemotherapy drug may influence the response (36, 47), but these clinical features do not help to predict the chemotherapy response. Using only clinical features gives a prediction with an AUC of only 0.523 ± 0.09 and combining clinical features with radiomic features does not improve the prediction as well. There are two possible reasons. First, the significance comparison is for groups, whereas response prediction involves individuals; features or parameters with significant differences are not necessarily discriminative nor do they always work for individual prediction (12). Second, the relation between clinical and radiomic features is more likely to be correlated than complementary; the radiomic features may represent the information underlying the clinical features and thus, make the latter redundant.

Whether combining clinical features with radiomic features improves the prediction is uncertain and specific to the task. For example, Velazquez et al. found that doing so substantially improved the predictive performance (AUC = 0.86) of EGFR mutation status, whereas using only clinical features gave a predictive model with an AUC of 0.81 (43). Moreover, Lou et al. found that models with both radiomic and clinical features were significantly better at predicting treatment failures than those with only radiomic features (21).

### Limitations and Future Work

The present study has limitations. First, our data set comprises CT images and treatment records of only 280 patients. Although overfitting was controlled, the sample size was relatively small. Second, the numbers of patients were unbalanced between adenocarcinoma and squamous cell carcinoma (228 *vs*. 52). Third, the type and dose of chemotherapy drug were not accounted. Finally, none of the predictive models were constructed using either deep learning or the hybrid method of deep learning and machine learning.

As future work, we will use a predictive model with overall survival as the prognostic endpoint. A deep convolutional neural network will be used to improve the predictive performance and the radiomic nomogram will help facilitate clinical applications (48–50). For a given NSCLC histological type and choice of chemotherapy drug (AP, GP, TP, or DP), a predictive response model may help oncologists choose the correct chemotherapy drug according to the patient’s histological type and pre-treatment CT images.

## Conclusion

The chemotherapy response of NSCLC patients can be predicted by a radiomic model based on machine leaning of pre-chemotherapy CT images. Several radiomic features differed significantly between the response and progression groups and could be used as imaging biomarkers to predict the chemotherapy response. The NSCLC tumors were more heterogeneous in CT images in the response group than in the progression group. This radiomic model with these imaging biomarkers could help to stratify NSCLC patients and make better treatment decisions, simply, non-invasively, and inexpensively.

## Data Availability Statement

The datasets presented in this article are not readily available because they must be approved by the Ethics Committee of Shengjing Hospital of Chinese Medical University. Requests to access the datasets should be directed to Shouliang Qi, (qisl@bmie.neu.edu.cn).

## Ethics Statement

The studies involving human participants were reviewed and approved by the ethics committee of Shengjing Hospital of China Medical University. Written informed consent for participation was not required for this study in accordance with the national legislation and the institutional requirements.

## Author Contributions

RC performed experiments and analyzed the data. SQ, XZ, and WQ proposed the idea, made discussions, composed the manuscript together with RC. YY collected and analyzed the data. JS directed the algorithm development and analyzed the data. All authors contributed to the article and approved the submitted version.

## Funding

This study was supported by the National Natural Science Foundation of China (Grant number: 82072008, 81671773, 61672146) and the Fundamental Research Funds for the Central Universities (Grant number: N2124006-3).

## Conflict of Interest

The authors declare that the research was conducted in the absence of any commercial or financial relationships that could be construed as a potential conflict of interest.
